# Aggregation Effects on Optimal Sensor Network Configurations with Distance-Dependent Noise

**DOI:** 10.3390/s25175381

**Published:** 2025-09-01

**Authors:** Russell Costa, Thomas A. Wettergren

**Affiliations:** Naval Undersea Warfare Center, Newport, RI 02841, USA

**Keywords:** distributed sensor network, aggregation, D-optimality, sensor configuration, optimal planning, source localization, environmental dependence

## Abstract

Optimizing sensor placement for the accurate localization of an uncertain source is crucial for a variety of distributed sensor network applications. To handle the uncertainty of the source locations, objective functions are typically written as an aggregation over a variety of plausible source locations. While prior research has explored how the resulting optimized sensor configurations correspond to the optimization over different objectives and various aggregations, the combined impact of both a complex noise environment and the choice of aggregation function for handling source uncertainty remains largely unexplored. This paper investigates this critical interplay. We demonstrate that incorporating distance-dependent environmental noise models reveals a strong dependence of the optimal sensor configuration on the aggregation method. This dependence affects diverse sensor types in differing ways, and this distinction is illustrated by examining both bearings-only and range-only sensors. We develop computational strategies for finding optimal configurations for each of these sensor types, and illustrate their distinctive features through canonical example problems for each type. The results underscore the importance of carefully considering both the environmental complexity and the aggregation approach when employing robust and reliable localization systems in practical applications.

## 1. Introduction

Distributed sensor networks are utilized for a wide range of applications at a variety of scales, ranging from wildlife tracking and weather observation to crowd monitoring and intruder detection. One of the most common goals of such systems is to localize the uncertain location of some disturbance, which is commonly known as the source localization problem.

In the design of these networks, necessary limitations on the number of available sensors means that the spatial pattern (or configuration) of the sensors has a large impact on the quality at which the localization can be performed. Hence, optimization of the sensor configuration is important for achieving maximum accuracy over the region of interest in these resource-constrained systems. Understanding how the characteristics of these optimal configurations depend upon sensor parameters, the environmental (noise) conditions, and sensor type is important for maintaining robust and reliable solutions to these planning problems.

Optimizing sensor placement is essential for maximizing localization accuracy in situations where the practical resource constraints limit the ability to otherwise flood a domain with sensors [1,2,3,4]. When there is no prior knowledge of the disturbance location, then the sensor placement problem becomes a problem of covering the region with sensors as effectively as possible. These sensor coverage problems are typically solved as some type of sensor packing problem [5], which is a specific form of the generalized problem of facility location coverage [6]. Within the domain of sensor coverage problems, the specific problem of achieving a certain level of performance in finding an uncertain disturbance is known as the Q-coverage problem [7]. While coverage is easily defined for any single disturbance location (i.e., sensors cover the location based on their relative positions and capabilities), the extension to an uncertain location requires some aggregation of the coverage performance over a range of possible disturbance locations [8].

We consider problems in which a set of sensors are to be fixed/positioned over an extended region in order to find and localize some object. The object is assumed to create a signal that can be received on passive sensors. The signal can take the form of a scent, a sound, a pressure wave, a visual disturbance, or any other phenomenon that is observable in a measurable way by the sensors that are being placed. Systems like this have been used for terrestrial wildlife monitoring [9], environmental monitoring [10], aerial vehicle (drone) monitoring [11], finding vehicles in varying terrain [12], and localizing robots and/or workers in manufacturing environments [13]. In all such cases, the sensors either measure the range to the source object or they measure the relative bearing to the object. Thus, we focus on range-only and bearings-only sensors that are deployed to cover an area to find and/or localize an object of interest.

To optimize these sensor configurations for coverage, a variety of optimization strategies have been employed. The main categories of these optimization strategies fall into four categories [14]: (i) genetic algorithms, (ii) computational geometry approaches, (iii) artificial potential fields, and (iv) particle-based nature-inspired optimizations. By far the most common of these in practice are genetic algorithms. In the application of genetic algorithms for the optimization of sensor configurations, the careful representation of a set of positions as a genetic chromosome is important for good algorithm convergence. Common approaches involve representing sensor positions as samples from a parameterized distribution function [15], limiting the positions of sensors to points on a grid [16], and permutation-preserving encodings [17]. As genetic algorithms only provide approximate solutions to the global optimization problem, coupling a genetic algorithm with a local optimization approach is a way to further improve performance [18]. Other successful approaches for optimizing sensor network configurations for coverage include simulated annealing [19] and ant colony optimization [20].

While coverage optimization is valuable in applications such as intruder detection, it does not necessarily provide the best configurations for applications where the localization of the disturbance source is desired. In these source localization problems, information theoretic performance metrics are used as optimization objectives instead of basic coverage. Numerous studies have addressed this optimization, employing various techniques and performance metrics [21,22,23,24]. Early work on these types of problems focused on analytical solutions for simplified scenarios, often utilizing geometric principles and the Cramer–Rao lower bound (CRLB) [21,25]. Other papers have shown that different optimality criteria, such as A-optimality, E-optimality, and D-optimality, offer distinct approaches to minimizing localization error, each with its own characteristics and tradeoffs [23,26,27,28]. Some of these early studies showed that the A-optimality, E-optimality, and D-optimality all lead to similar optimal configuration patterns [28]. Among these choices of optimality criteria, the D-optimality, which maximizes the determinant of the Fisher Information Matrix (FIM), is particularly attractive for source localization. For instance, maximizing the D-optimality measure minimizes the volume of the uncertainty ellipsoid, which directly reduces the overall uncertainty in the source location estimate [29]. This property makes D-optimality well-suited as a performance metric (and optimization objective) for scenarios where the potential source location is certain, but this measure must be aggregated over varying potential source locations to account for uncertainty.

For a specific location of a potential disturbance/source, the relationship between sensor position and the source location can be optimized by using these performance metrics evaluated for the specific sensor modality [30,31]. However, practical scenarios frequently involve uncertainty in the source location, demanding more complex optimization approaches. This uncertainty is typically handled by using probability density functions [22] or through grid-based representations [32], often integrating one of the previously-mentioned performance metric functions over all of the potential source locations. Such optimizations provide a configuration of the sensor placements that is specific to the sensor modality and the problem geometry [33,34]. In addition, the shape of the boundary of the region that is known to contain the source of interest (called the region of interest) can significantly influence the optimal sensor configuration due to boundary effects [32]. This effect is observed as the optimal sensor configuration taking on specific patterns that are tied to the particular shape of the region of interest. In this paper, we represent the uncertainty in source point location as a two dimensional uniform probability mass function contained within a square region of interest.

In addition to the information theoretic performance model and the shape of the region of interest, the noise inherent in the region has an impact on the sensor performance, and hence the optimal configurations. While there have been many advancements in optimal sensor placement in idealized scenarios, a critical gap exists in understanding the combined effects of noise models and aggregation methods. Most existing studies assume distance-independent noise for analytical tractability [24,35], but many practical distributed sensing scenarios involve signal attenuation with distance [36,37], leading to distance-dependent covariance structures [38]. These distance-dependent noise models have been used to model sensor network applications such as those with varying levels of vegetation in terrestrial problems [12], sensing through clutter in industrial environments [13], and sensing AUV’s underwater [39]. In developing optimal configurations in such applications, the aggregation of performance information from various potential source locations into a single objective function must be performed to find an optimal sensor configuration for an unknown source location. The consideration of choosing a specific form of aggregation function remains largely overlooked, as only nominal aggregations are typically used in these applications. While [32] briefly examined this aspect for bearings-only localization under distance-independent noise, a comprehensive analysis considering both distance dependence and a diverse set of potential aggregation approaches for different sensor types is lacking. This paper addresses this gap by investigating the interplay between distance-dependent noise covariance structures and aggregation function choice. We demonstrate a previously unobserved dependence that impacts optimal sensor configurations across various sensor types, highlighting the need for careful consideration of both factors in practical localization system design.

In Section 2, we describe the concept of a performance surface, which visually represents the localization accuracy of a particular sensor configuration, and derive the FIM for two sensor paradigms of interest. In Section 3, we construct several numerical examples that allow us to quantify the effect of different aggregation choices, where for each choice of aggregation we solve a numerical optimization problem. We analyze and discuss the features of the optimal configurations for different aggregations and sensor schemes and the impact of varying levels of the noise range-dependence on those configurations. Finally, in Section 4, we share the conclusions of our study with a note to future work.

## 2. Information-Based Measures of Localization Accuracy

We consider the placement of *N* sensors within a bounded region $\Omega \subset R^{2}$. Let the location of the ith sensor be given by $s_{i}\in \Omega$ for i=1,2,…,N, where the specific coordinates of the sensor location are given as $s_{i}=\left[x_{i},y_{i}\right]$. Hence the sensor network is represented by the set of sensor locations (or sensor configuration) $S={\left\{s_{i}\right\}}_{i=1}^{N}$. We represent an arbitrary source location θ∈Ω by its coordinates $\theta =[x^{\left(\theta \right)},y^{\left(\theta \right)}]$, where the superscript (θ) is used to distinguish a source coordinate pair from the sensor coordinate pairs. For an uncertain source location, the quality with which a given sensor network can observe that location is given by the information measure f(S,θ;η), which is parameterized by a set of environmental condition parameters η that encompass the noise characterization.

For any sensor network configuration S and a single source location $\theta =\theta_{j}$, we derive a closed-form expression for the FIM of the source location parameters. The determinant of this 2×2 FIM (also called the DFIM), provides a scalar measure of sensor network performance that can be used as the information measure $f(S,\theta_{j};\eta )$. In particular, this FIM-based information measure is inversely proportional to the volume of uncertainty in the source location estimate. Given a set of potential source locations $\left\{\theta_{1},\theta_{2},\ldots ,\theta_{M}\right\}$, we calculate such an information measure (e.g., the determinant of the FIM or DFIM) for a fixed sensor configuration S that is conditioned on each possible source location $\theta_{j}$. If the uncertain plausible source locations $\theta_{j}$ span the domain Ω, then this set of DFIM values represents a performance surface over Ω, which can be visualized as an image or contour plot, reflecting the localization accuracy for a given sensor configuration S. Optimizing the DFIM is known as D-optimality in the design of experiments community. D-optimality has been shown to be beneficial for configuring sensor network placements in closed domains [40]. This is particularly beneficial when sensors are positioned around a single target location [41]. While some early studies have shown that A-optimality, D-optimality and E-optimality can lead to similar configurations in certain cases [28], it has been recently pointed out that A-optimality leads to better results when the statistics of the noise are unknown, yet D-optimality provides better results when the statistics of the noise are known [42]. Hence, we only consider D-optimality in this study, as our focus is on how the particular nature of the noise model (along with aggregation) impacts the optimal configurations.

When the goal is to determine the optimal configuration S of the sensor network (i.e., the configuration that provides the best overall localization accuracy), these individual localization accuracies for specific locations must be aggregated into a scalar objective value. This scalar objective value is then utilized within existing numerical optimization approaches to find the optimal configuration. The goal of this optimization is to find the sensor configuration S that yields the optimal performance surface with respect to the chosen aggregation function for a given environmental condition (as represented by the noise uncertainty parameters).

### 2.1. Fisher Information Measure for Range-Only Sensors

The first type of sensing mode we consider for a sensor network is a range-only sensor. Range-only sensors are used in many localization contexts and are closely related to time-difference-of-arrival (TDOA) sensor applications. Let $\theta =\left[x^{\left(\theta \right)},y^{\left(\theta \right)}\right]$ correspond to an arbitrary, fixed source location. Recall that *N* is the number of sensors to be deployed and $s_{i}=\left[x_{i},y_{i}\right]$ is the ith sensor location in Ω. The actual range (distance) between the ith sensor and the arbitrary source location is defined as

(1) $$\begin{array}{c} r_{i}=\sqrt{{\left(x_{i}-x^{\left(\theta \right)}\right)}^{2}+{\left(y_{i}-y^{\left(\theta \right)}\right)}^{2}}. \end{array}$$

The measurement equation for the observed range $z_{i}$ is given as

(2) $$\begin{array}{c} z_{i}=\left(1+\omega_{i}\right)r_{i}+\nu_{i}, \end{array}$$

where $\omega_{i}$ and $\nu_{i}$ (the multiplicative and additive noise components, respectively) are independent and identically distributed (i.i.d.) random variables that are distributed as $N(0,{\sigma_{\omega }}^{2})$ and $N(0,{\sigma_{\nu }}^{2})$, respectively [43]. The range “measurement” is typically mapped via some time-of-flight measurement or is based on the received signal strength measured from a known source. The assumption of this measurement equation assumes that the mapping into the range value results in this form of random variable. This is the most basic form of noise model that captures the features of distance-dependence. More complicated noise models may be appropriate for specific applications and are a subject of future work.

The range-only sensor measurement equation (given by Equation (2)) maps to the observation $z_{i}$ being distributed as $N(r_{i},{\sigma_{i}}^{2})$, where

(3) $$\begin{array}{c} {\sigma_{i}}^{2}=\gamma {r_{i}}^{2}+{\sigma_{\nu }}^{2} \end{array}$$

is the observation variance. In this noise model $\gamma ={\sigma_{\omega }}^{2}$ is the distance-dependent noise parameter for range-only localization and ${\sigma_{\nu }}^{2}$ is the distance-independent contribution to sensor noise. Since the sensor observations are assumed to be independent of one another, the joint probability density function (PDF) of the observations for all of the sensors is the product

(4) $$\begin{array}{c} p\left(Z|\theta \right)=\prod_{i=1}^{N}p\left(z_{i}|x^{\left(\theta \right)},y^{\left(\theta \right)}\right), \end{array}$$

where $Z={\left[z_{1},z_{2},\ldots ,z_{N}\right]}^{T}$ is the vector of observed ranges from the *N* sensors and θ represents a particular source location. In the joint sensor PDF given by Equation (4), the individual sensor PDFs are given as

(5) $$\begin{array}{c} p\left(z_{i}|x^{\left(\theta \right)},y^{\left(\theta \right)}\right)=\frac{1}{\sqrt{2\pi {\sigma_{i}}^{2}}}\exp\left(-\frac{{\left(z_{i}-r_{i}\right)}^{2}}{2{\sigma_{i}}^{2}}\right). \end{array}$$

where we recall that $z_{i}$ is the observed range from the sensor located at $s_{i}=\left[x_{i},y_{i}\right]$.

Thus, the joint PDF in Equation (4) is a multivariate Gaussian PDF with the mean vector $\mu ={\left[r_{1},r_{2},\ldots ,r_{N}\right]}^{T}$ and covariance matrix $C=\mathrm{diag}\left(\sigma_{1}^{2},\sigma_{2}^{2},\ldots \sigma_{N}^{2}\right)$. Here, diag(·) forms a matrix with the arguments on the diagonal and zeros everywhere else. Let the 2×2 matrix *F* represent the FIM for the sensor observations. Given the joint PDF of the observations from individual sensors whose PDFs are in the form of Equation (5), the components $F_{lk}$ of the FIM (where the subscripts *l* and *k* denote the row and column of the FIM, respectively) are written as

(6) $$\begin{array}{cc} F_{lk} & =-E\left[\frac{\partial^{2}\ln\left(p\left(Z|\theta \right)\right)}{\partial \theta_{l}\partial \theta_{k}}\right]\\ \; & ={\left[\frac{\partial \mu (\theta )}{\partial \theta_{l}}\right]}^{T}C^{-1}\left(\theta \right)\left[\frac{\partial \mu (\theta )}{\partial \theta_{k}}\right]\\ \; & \;+\frac{1}{2}\mathrm{tr}\left[C^{-1}\left(\theta \right)\frac{\partial C(\theta )}{\partial \theta_{l}}C^{-1}\left(\theta \right)\frac{\partial C(\theta )}{\partial \theta_{k}}\right], \end{array}$$

where $\theta_{l}=x^{\left(\theta \right)}$ and $\theta_{k}=y^{\left(\theta \right)}$ are the coordinate components of θ, E[·] is the expectation operator, and tr[·] is the matrix trace operator [44]. Let $\phi_{i}$ be the bearing angle of the arbitrary source location $\theta =\left[x^{\left(\theta \right)},y^{\left(\theta \right)}\right]$ to the ith sensor $s_{i}=\left[x_{i},y_{i}\right]$, such that

(7) $$\begin{array}{c} \phi_{i}=\tan^{-1}\left(\frac{x_{i}-x^{\left(\theta \right)}}{y_{i}-y^{\left(\theta \right)}}\right). \end{array}$$

Then the FIM in this case is explicitly written as

(8) $$\begin{array}{c} F=\sum_{i=1}^{N}g_{i}\left(\begin{array}{cc} \cos^{2}\phi_{i} & \cos\phi_{i}\sin\phi_{i}\\ \cos\phi_{i}\sin\phi_{i} & \sin^{2}\phi_{i} \end{array}\right), \end{array}$$

where $g_{i}=\left(\sigma_{i}^{2}+2\gamma^{2}r_{i}^{2}\right)/\sigma_{i}^{4}$.

Each element of the FIM matrix *F* in Equation (8) contributes to the amount of information that a given sensor configuration S yields from a source at a fixed location $\theta =\left[x^{\left(\theta \right)},y^{\left(\theta \right)}\right]$. Generally, the FIM (when evaluated at the true value to be estimated) gives a measure of the best possible estimate of location yielded by a given sensor configuration, assuming an unbiased estimator. A higher value of Fisher information implies an improved lower bound on the variance of any unbiased estimator. This suggests that higher FIM values achieve a more precise (in terms of lower variance) estimate of the parameter θ. Hence, the data obtained is more informative about θ. As a scalar measure of the overall information, the determinant of the FIM (or DFIM) is adopted for range-only source localization, $D_{F_{RO}}$, which is given as follows:

(9) $$\begin{array}{c} D_{F_{RO}}\left(S,\theta ;\eta \right)=\det\left(F\right)=\frac{1}{2}\sum_{i=1}^{N}\sum_{j=1}^{N}g_{i}g_{j}\sin^{2}\phi_{ij}, \end{array}$$

where $\phi_{ij}=\phi_{i}-\phi_{j}$. The DFIM of Equation (9) provides a scalar representation of the amount of information that the sensor configuration S yields from a source at θ using a range-only sensing modality.

### 2.2. Fisher Information Measure for Bearings-Only Sensors

The second type of sensing mode we consider for a sensor network is a bearings-only sensor. Recall that $\theta =\left[x^{\left(\theta \right)},y^{\left(\theta \right)}\right]$ is a fixed arbitrary source location in the region of interest $\Omega \in R^{2}$. As in the case of range-only sensors, we have *N* as the number of sensors to be placed in a collaborative configuration as a sensor network S. The position of the *i*th sensor is given by $s_{i}=\left[x_{i},y_{i}\right]$ and the bearing angle of the source point θ to the sensor at location $s_{i}$ is given by $\phi_{i}$, as in Equation (7). The measurement equation for the observed bearing angle $\beta_{i}$ for the bearings-only localization is then written as

(10) $$\begin{array}{c} \beta_{i}=\phi_{i}+\xi_{i}, \end{array}$$

where $\xi_{i}$ is a Gaussian random variable distributed as $N(0,\sigma_{i}^{2})$. The measurement of bearing typically requires an array of smaller sensor elements that estimate the bearing through some maximization process over a discrete set of potential time delays that coincide with potential angles of arrival. By assumption, the measurement Equation (10) captures the statistics of such a process and hence represents the uncertainty in the bearing estimate. We assume that the variance of the bearings-only sensor has a similar distance-dependence as for the range-only sensor of the previous section (see Equation (3)), such that

(11) $$\begin{array}{c} \sigma_{i}^{2}=\gamma r_{i}^{2}+\sigma_{\nu }^{2}, \end{array}$$

where γ is the distance-dependent noise parameter and $\sigma_{\nu }^{2}$ is the distance-independent contribution to sensor noise [43]. As all of the sensors in the bearings-only case are assumed to be independent, the joint PDF of the observations $\beta_{i}$, i=1,2,…,N is a multivariate Gaussian PDF of the form

(12) $$\begin{array}{c} p\left(B|\theta \right)=\prod_{i=1}^{N}p\left(\beta_{i}|x^{\left(\theta \right)},y^{\left(\theta \right)}\right), \end{array}$$

with $B={\left[\beta_{1},\beta_{2},\ldots ,\beta_{N}\right]}^{T}$ representing the vector of observed bearings, each of which has the measurement model of Equation (10). The individual sensor PDFs in the joint PDF of Equation (12) are given by

(13) $$\begin{array}{c} p\left(\beta_{i}|x^{\left(\theta \right)},y^{\left(\theta \right)}\right)=\frac{1}{\sqrt{2\pi {\sigma_{i}}^{2}}}\exp\left(-\frac{{\left(\beta_{i}-\phi_{i}\right)}^{2}}{2{\sigma_{i}}^{2}}\right), \end{array}$$

where we recall that $\beta_{i}$ is the observed bearing from the sensor located at $s_{i}=\left[x_{i},y_{i}\right]$.

As the joint PDF in Equation (12) is a multivariate Gaussian PDF with the mean vector $\mu ={\left[\phi_{1},\phi_{2},\ldots ,\phi_{N}\right]}^{T}$ and covariance matrix $C=\mathrm{diag}\left(\sigma_{1}^{2},\sigma_{2}^{2},\ldots \sigma_{N}^{2}\right)$, it is clear that the FIM for the sensor network with bearings-only sensors can be derived from Equation (6) by replacing p(Z∣θ) with p(B∣θ). Each term in the FIM is written as in Equation (14) to denote the two terms as shown in Equation (6). The first term is dominated by the derivative of the observation mean and the second term includes the derivative of the distance-dependent covariance matrix, as shown by

(14) $$\begin{array}{c} F_{lk}=F_{lk}^{\left(A\right)}+F_{lk}^{\left(B\right)}, \end{array}$$

where

(15) $$\begin{array}{c} F_{lk}^{\left(A\right)}={\left[\frac{\partial \mu (\theta )}{\partial \theta_{l}}\right]}^{T}C^{-1}\left(\theta \right)\left[\frac{\partial \mu (\theta )}{\partial \theta_{k}}\right] \end{array}$$

and

(16) $$\begin{array}{c} F_{lk}^{\left(B\right)}=\frac{1}{2}\mathrm{tr}\left[C^{-1}\left(\theta \right)\frac{\partial C(\theta )}{\partial \theta_{l}}C^{-1}\left(\theta \right)\frac{\partial C(\theta )}{\partial \theta_{k}}\right]. \end{array}$$

It is straightforward to show that the terms of the FIM for the bearings-only case are thus given by the following:

(17) $$\begin{array}{c} F_{11}=\sum_{i=1}^{N}\frac{\sin^{2}\phi_{i}}{\sigma_{i}^{2}r_{i}^{2}}+2\gamma^{2}\sum_{i=1}^{N}\frac{r_{i}^{2}\cos^{2}\phi_{i}}{\sigma_{i}^{4}}, \end{array}$$

(18) $$\begin{array}{c} F_{22}=\sum_{i=1}^{N}\frac{\cos^{2}\phi_{i}}{\sigma_{i}^{2}r_{i}^{2}}+2\gamma^{2}\sum_{i=1}^{N}\frac{r_{i}^{2}\sin^{2}\phi_{i}}{\sigma_{i}^{4}}, \end{array}$$

(19) $$\begin{array}{c} F_{12}=F_{21}=-\sum_{i=1}^{N}\frac{\cos\phi_{i}\sin\phi_{i}}{\sigma_{i}^{2}r_{i}^{2}}+2\gamma^{2}\sum_{i=1}^{N}\frac{r_{i}^{2}\sin\phi_{i}\cos\phi_{i}}{\sigma_{i}^{4}}. \end{array}$$

The determinant of the FIM (or DFIM) for the bearings-only source localization, $D_{F_{BO}}$, is then given as

(20) $$\begin{array}{c} D_{F_{BO}}\left(S,\theta ;\eta \right)=F_{11}F_{22}-F_{12}^{2}, \end{array}$$

where $F_{11}$, $F_{22}$, and $F_{12}$ are given by Equations (17)–(19), respectively. The DFIM of Equation (20) provides a scalar representation of the amount of information that the sensor configuration S yields from a source at θ using a bearings-only sensing modality.

### 2.3. Aggregation into a Single Measure of Performance

The information measures of the previous sections (e.g., $D_{F_{RO}}$ from Equation (9) and $D_{F_{BO}}$ from Equation (20)) provide functions f(S,θ;η) that represent the performance of a sensor configuration S against a single uncertain source location θ in an environment with the noise parameterized by η. For practical problems, we must consider the performance over a range of potential source locations. One way to do this is to consider a set of equally likely “plausible” source locations $\left\{\theta_{1},\theta_{2},\ldots ,\theta_{M}\right\}$. To find optimal sensor configurations across this entire set of locations, we aggregate the information measures f(S,θ;η) that are associated with each plausible location into a single performance metric. In this paper, we utilize the generalized mean family of functions to perform such an aggregation. These functions are well-studied and possess numerous mathematical properties that provide flexibility in how we approach optimizing localization accuracy over the entire region of interest.

The generalized mean is defined as follows [45]:

**Definition** **1** (Generalized Mean)**.** *Consider $x_{1},x_{2},\ldots ,x_{n}$ as n arbitrary positive numbers, then the generalized mean is given as*

(21) $$\begin{array}{c} \mu_{r}\left(x_{1},x_{2},\ldots ,x_{n}\right)={\left(\frac{1}{n}\sum_{j=1}^{n}x_{j}^{\alpha }\right)}^{1/\alpha } \end{array}$$

*where the parameter α controls the type of mean function.*

Note that this definition is for equal weights, but, in general, any weighting is possible by multiplying each $x_{j}^{\alpha }$ by a weight $w_{j}\geq 0$ with $\sum_{j}w_{j}=1$. Common examples for means, such as the geometric, harmonic, and arithmetic mean, correspond to α=0, α=−1, and α=1, respectively. Also, the minimum function and maximum function correspond to α=−∞ and α=+∞, respectively.

The combination of information measures using generalized means is given by the aggregation of information, defined as follows [32]:

**Definition** **2** (Aggregation of Information)**.** *The aggregation of information for a sensor network S with M possible source locations $\theta_{1},\theta_{2},\ldots ,\theta_{M}$ is written as*

(22) $$\begin{array}{c} J\left(S\right)={\left(\frac{1}{M}\sum_{j=1}^{M}{\left[f(S,\theta_{j};\eta )\right]}^{\alpha }\right)}^{1/\alpha } \end{array}$$

*where $f\left(S,\theta_{j};\eta \right)=D_{F_{RO}}$ for the range-only sensors and $f\left(S,\theta_{j};\eta \right)=D_{F_{BO}}$ for the bearings-only sensors.*

This aggregation provides a single performance value for a given sensor configuration, considering all possible, equally likely source locations. For a chosen value of α, we aim to maximize this performance function with respect to the sensor positions. A key property of the generalized mean is that, for a fixed set of positive values, its output is bounded by the minimum and maximum of those values across all possible α values. This has a significant spatial interpretation. When α=−∞, the aggregation selects the minimum information measure among all locations, meaning the optimization focuses on maximizing the worst-case estimation accuracy and prioritizing improved performance for the totality of source locations. Conversely, when α=+∞, the aggregation selects the maximum information measure. Optimization then focuses on maximizing performance for some locations, potentially arbitrarily at the expense of others, aiming for exceptional performance in a smaller subset of scenarios. Therefore, varying α allows us to tune the optimization strategy between prioritizing worst-case individual location performance (α=−∞) and best-case individual location performance (α=+∞).

## 3. Numerical Optimization Results

To investigate the interplay between the method of aggregation for source location uncertainty and the level of distance-dependent noise, we construct several numerical examples. We construct examples where the expected optimal configurations will be symmetric for fixed γ, then run them over a span of α values corresponding to different aggregations. Recall that the method of aggregation is given by the parameter α in Equation (22), and the level of distance-dependent noise is given by the parameter γ in the noise models of Equations (3) and (11). All of the cases that follow utilize the following region of interest and grid of plausible source locations. Define $\Omega \subset R^{2}$ as a square where each axis, both vertical and horizontal, spans −1.5 to 1.5 units. A set of *M* = 10,000 possible source locations is used, where the source locations are placed at a 100×100 grid that is equally spaced in both dimensions. This resolution was selected such that the grid is dense enough that the results become similar to those for a continuum of possible source locations but sparse enough to provide reasonable computational time. It is assumed that all source locations are equally likely, so a uniform weighting is applied to the summation in the aggregation formula. The optimization problem to be solved is stated as

(23) $$\begin{array}{c} S^{*}=\arg\max_{S}J\left(S\right)\;\mathrm{such}\;\mathrm{that}\;S\in \Omega , \end{array}$$

where J(S) is a scalar measure from aggregation of the information measures with respect to all possible source locations (using the form of Equation (22)). This optimization problem represents a nonlinear objective function J(S) with simple bound constraints on the sensor location variables S. We numerically solve the optimization problem (23) by using the Sequential Quadratic Programming (SQP) method within the functional solver fmincon in the MATLAB version 2019a optimization toolbox.

### 3.1. Optimal Configurations for Range-Only Sensors

The first example solves the optimization in Equation (23) using the DFIM for range-only sensors ($D_{F_{RO}}$) for optimally placing sensors S in a region Ω, as defined above. The objective function in this case is given using the DFIM of Equation (9), with aggregation in the form of Equation (22) as

(24) $$\begin{array}{c} J\left(S\right)={\left(\frac{1}{M}\sum_{j=1}^{M}{\left[D_{F_{RO}}\left(S,\theta ;\eta \right)\right]}^{\alpha }\right)}^{1/\alpha }. \end{array}$$

**Figure 1:**
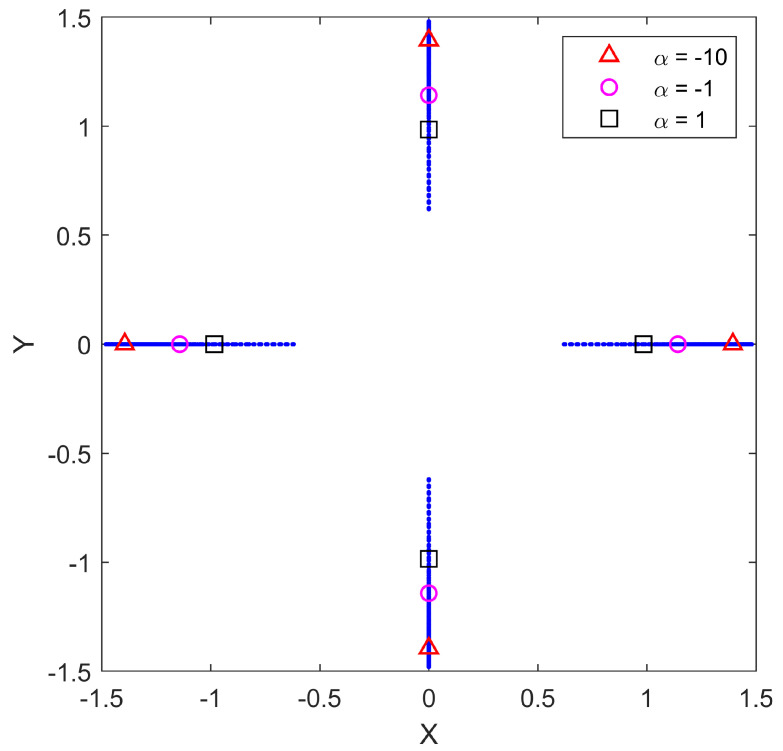
Optimal sensor positions S for N=4 range-only sensors from α=−20 (outside points) to α=5 (inside points) for γ=0.5. Red triangles show the sensor locations for α=−10, magenta circles show the sensor locations for α=−1, and black squares show the sensor locations for α=1.

For this example, the number of sensors in S is fixed as N=4, and the optimization is performed for various choices of aggregation function (parameterized by α), with varying levels of distance-dependence of the measurement noise (parameterized by γ) and with the additive noise component $\sigma_{\nu }^{2}=1$. The goal is to quantify the effect of different choices of aggregation function on the optimal configuration of sensors for varying amounts of distance-dependent noise. By fixing γ and then solving the optimization for a variation of α values, we obtain a family of solutions, each an optimal configuration with respect to a given α. In the range-only sensor paradigm, the optimal configuration of N=4 sensors takes the form of a diamond. Since all of the optimal configurations are of the same shape, we can then quantify the dependence on aggregation through a single measure of the spacing. In Figure 1, the geographical locations of the optimal positions S are shown for each value of α for the specific value of γ=0.5. From this figure, it is clear that the configuration shape remains the same for all cases studied, as only the size of the configuration changes with α. Along with the optimal positions for the entire span of α values, we show more prominently the optimal positions for α=−10, α=−1, and α=1, respectively. This gives a perspective on the sensitivity of the optimal configurations as a function of α. Similar results were found for each value of γ. We calculate the spacing measure of each optimal sensor configuration by forming the convex hull of the sensor points and averaging the length of all four sides. This number will be referred to as the measure of the spacing of a given configuration.

To solve for the family of optimal sensor configurations for fixed γ, we first run the SQP solver for the optimization problem of Equation (23) using the J(S) from the objective function in Equation (24) with a large number of random starts (n=100) for an aggregation choice of α=−20. We choose the optimal configuration as the solution corresponding to the maximum among the 100 independent SQP solutions. Using the optimal configuration corresponding to α=−20, we increase α in small increments of 0.1 (i.e., from α={−20.0,−19.9,−19.8,…}) and run the SQP solver recursively (once for each value of α), where for each increment the optimal sensor locations from the prior increment are used as the initial variable values for the optimization. In this way, we find a family of solutions recursively by increasing α from −20 to 5. Conversely, we repeat this procedure starting with n=100 random starts for an aggregation choice of α=5 and decreasing in small increments recursively to α=−20. This provides two optimization solutions for each value of the aggregation parameter α, which may or may not be the same due to the local nature of the SQP optimization. For each value of α, we choose the optimal sensor configuration corresponding to the pointwise maximum between the two optimization solutions. While there is no guarantee that the SQP solver will yield a globally optimal solution, this exhaustive approach, coupled with the symmetry and relatively small dimensionality of the example problems, is expected to yield a global optimum with a high degree of certainty.

**Figure 2:**
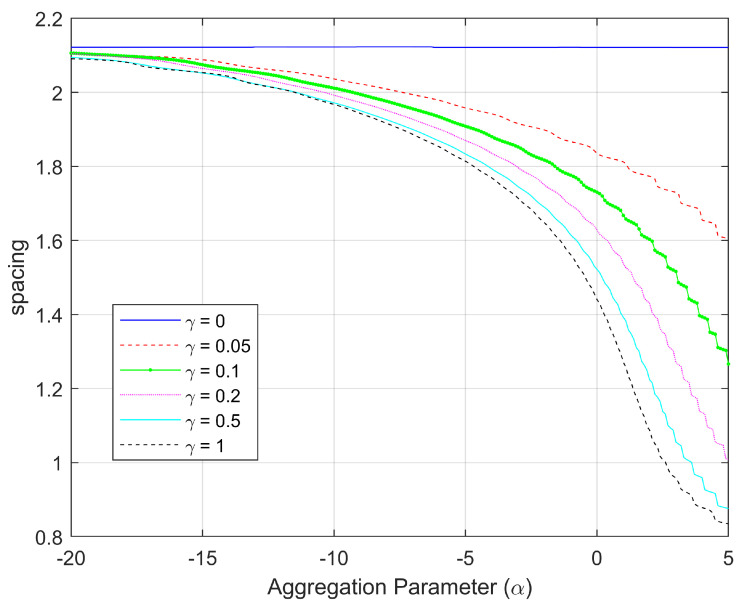
The spacing measure associated with the optimal configurations for N=4 range-only sensors as a function of aggregation parameter α. The different curves reflect different amounts of distance-dependent noise given by γ.

For this example, the spacing measure of each optimal configuration (for specific fixed γ) is plotted as a function of α in the family of curves shown in Figure 2. It is clear from Figure 2 that the spacing of the optimal configurations is heavily dependent on the amount of distance-dependence in the environmental noise (given by γ). When γ=0 (i.e., no distance-dependence), there is no change in the optimal configuration by varying the aggregation, but even a modest value of γ=0.05 causes the effect of the choice of aggregation parameter to increase significantly. Figure 2 further shows that this dependence on aggregation becomes more prominent for even larger values of γ. For the well-known mean aggregations, such as harmonic (α=−1), geometric (α=0), and arithmetic (α=1), we see that the spacing measure is most robust to environmental changes for the harmonic mean. More generally, we observe that the optimal configurations corresponding to α=−20 change very little with variation in γ, making them the most robust to changes in distance-dependent noise. Note that α=−20 is starting to get close to an optimization approach that would aim to maximize the minimum DFIM over the 10,000 source locations of interest (which would occur as α→−∞).

**Figure 3:**
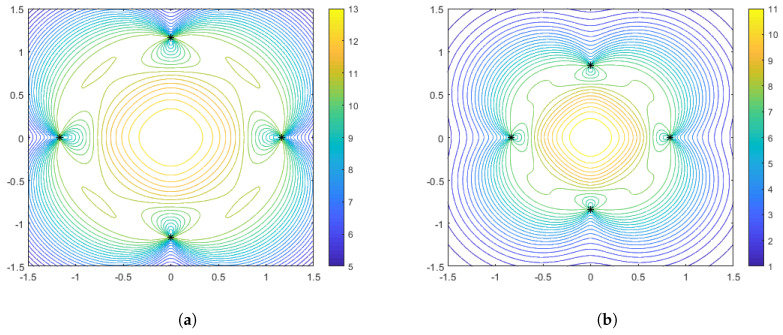
Optimal configurations for N=4 range-only sensors with aggregation of α=1. Color contours represent the DFIM values from Equation (9) plotted over various values of source location θ. The left plot (**a**) is for weak distance-dependent noise (γ=0.05), and the right plot (**b**) is for strong distance-dependent noise (γ=0.5).

**Figure 4:**
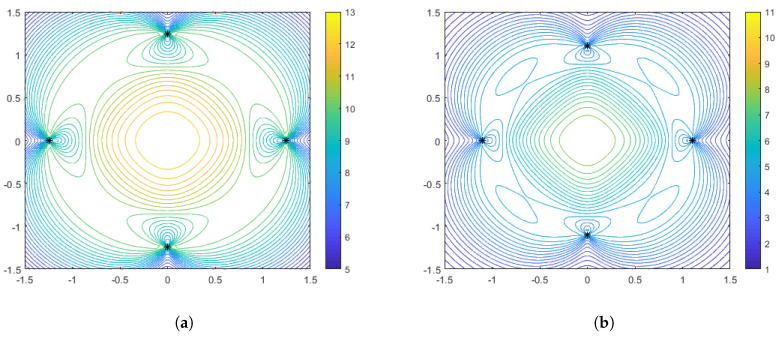
Optimal configurations for N=4 range-only sensors with aggregation of α=−1. Color contours represent the DFIM values from Equation (9) plotted over various values of source location θ. The left plot (**a**) is for weak distance-dependent noise (γ=0.05), and the right plot (**b**) is for strong distance-dependent noise (γ=0.5).

**Figure 5:**
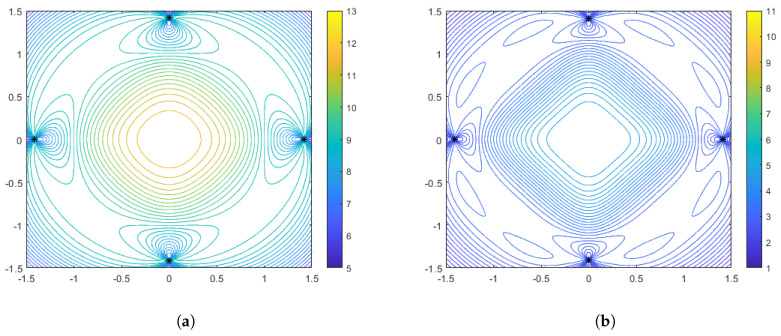
Optimal configurations for N=4 range-only sensors with aggregation of α=−10. Color contours represent the DFIM values from Equation (9) plotted over various values of source location θ. The left plot (**a**) is for weak distance-dependent noise (γ=0.05), and the right plot (**b**) is for strong distance-dependent noise (γ=0.5).

In Figure 3, Figure 4 and Figure 5 we illustrate the features of the performance surface relative to some select points on the optimal spacing curves. Figure 3 shows the optimal configurations and corresponding performance surface for α=1 (arithmetic mean), for both weak distance-dependence (left) and strong distance-dependence (right). Figure 4 and Figure 5 show the same for α=−1 and α=−10, respectively. We observe several notable features that are consistent with the spacing curves. First, for the weaker distance-dependence we see that there are much smaller changes in the spacing of the optimal configurations than in the stronger distance-dependent noise case. Subsequently, the variations in the features of the performance surface are more prevalent in the stronger distance-dependence case. In particular, the dynamic range of the DFIM is significantly larger in the stronger distance-dependence case than in the weaker distance-dependence case for the α=−1 and α=1 cases, shown in Figure 3 and Figure 4, respectively. This is because the spacing differences are affected by the larger $\sigma_{i}$ (note that larger γ leads to larger $\sigma_{i}$ per Equation (3)), with the optimal configurations for larger γ being tighter in the center of the region. The performance surfaces shown in Figure 4 for the α=−1 case are comparable in flatness for the two values of γ, with the overall DFIM levels lower in the higher γ case. Note that for all of these plots the dynamic range in the colormaps were tuned to the maximum and minimum DFIM for a fixed γ across the variations in aggregation parameter. This is so the performance surface features could be prominently viewed as a function of aggregation.

### 3.2. Optimal Configurations for Bearings-Only Sensors

The second example solves the optimization in Equation (23) using the DFIM for bearings-only sensors ($D_{F_{BO}}$) for optimally placing sensors S in a region Ω as defined above. The objective function in this case is given using the DFIM of Equation (20), with aggregation in the form of Equation (22) as

(25) $$\begin{array}{c} J\left(S\right)={\left(\frac{1}{M}\sum_{j=1}^{M}{\left[D_{F_{BO}}\left(S,\theta ;\eta \right)\right]}^{\alpha }\right)}^{1/\alpha }. \end{array}$$

**Figure 6:**
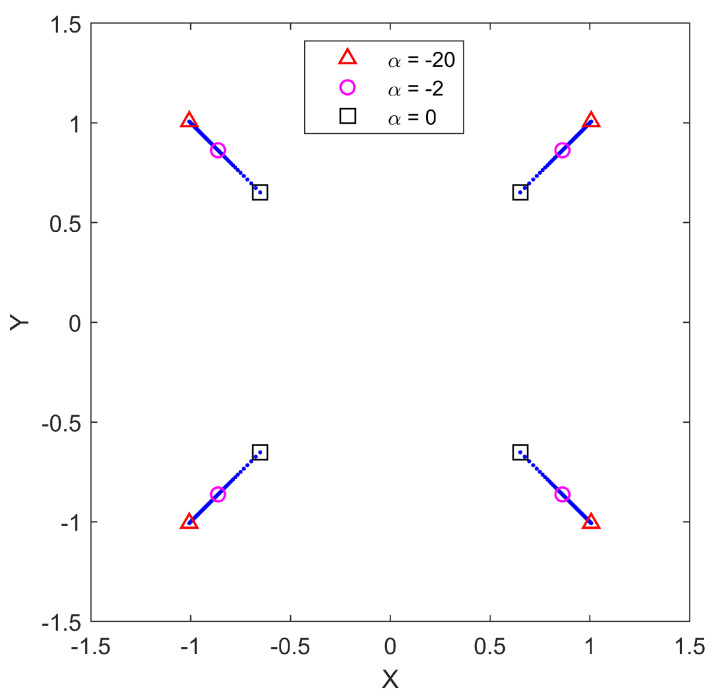
Optimal sensor positions S for N=4 bearings-only sensors from α=−20 (outside points) to α=0 (inside points) for γ=0.1. Red triangles show the sensor locations for α=−20, magenta circles show the sensor locations for α=−2, and black squares show the sensor locations for α=0.

As with the range-only example, the number of sensors in S is fixed as N=4, and the optimization is performed for various choices of aggregation function (parameterized by α) with varying levels of distance-dependence of the measurement noise (parameterized by γ) with the additive noise component $\sigma_{\nu }^{2}=0.5$. Once again, the goal is to quantify the effect of different choices of aggregation function on the optimal configuration of sensors for varying amounts of distance-dependent noise. In this case, the optimization is limited to aggregation values in the range of α=−20 to α=0. As discussed in [32], the optimization framework is not viable for α>0 in the bearings-only case. This is because the FIM is numerically unstable when any sensor gets too close to any particular source position. Thus, optimizing the numerical objective in equation (25) results in nonsensical solutions with unbounded objective evaluations. Figure 6 shows the geographical locations of the optimal positions S for each value of α for the specific value of γ=0.1. From this figure, it is seen that for the bearings-only case, the optimal configurations are box shaped, with the smallest box corresponding to the geometric mean (α=0) and larger boxes corresponding to smaller values of α. To highlight the sensitivity of the optimal configurations to the aggregation parameter α, we explicitly plot the optimal configurations for α=−20, α=−2, and α=0, respectively. It is clear that the optimal sensor positions are more sensitive for larger magnitude α values (those closer to −∞). We note that the shape remains the same, although the size (and possibly orientation) of the configuration may change with changes to α and/or γ.

**Figure 7:**
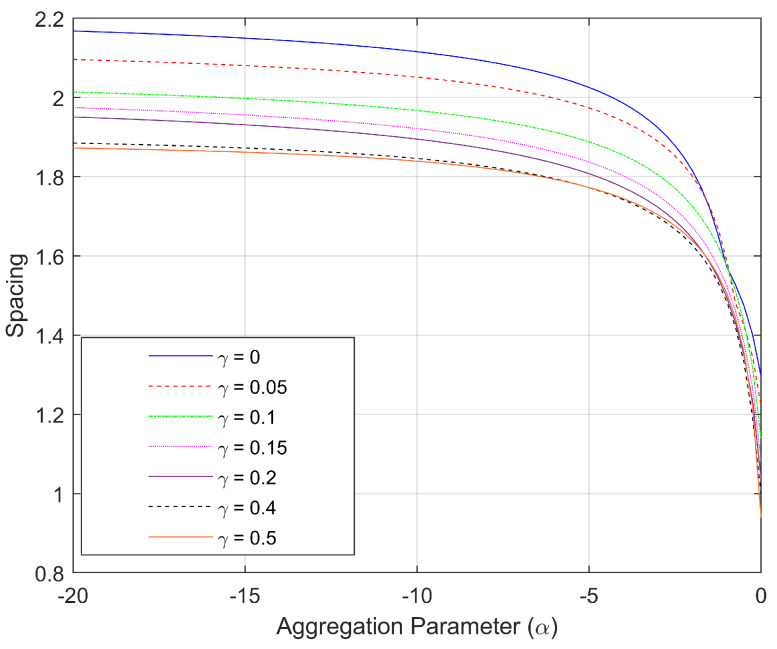
The spacing measure associated with the optimal configurations for N=4 bearings-only sensors as a function of aggregation parameter α. The different curves reflect different amounts of distance-dependent noise given by γ.

In Figure 7, we plot the spacing measure of each optimal configuration (for specific γ) for bearings-only sensors as a function of the aggregation parameter α. Unlike the range-only case, for this bearings-only case, the spacing of the optimal configurations depend on α even when there is no distance-dependent noise (i.e., γ=0). This is due to an intrinsic distance-dependence of the FIM components shown in Equations (17)–(19) with the $r_{i}^{2}$ in the denominator of the first terms. In addition, although the optimal spacing decreases rapidly for α>−4, those corresponding configurations are more robust to changes in the distance-dependent noise γ than those configurations with α<−4. This is the opposite of what was observed in the range-only sensor paradigm (see Figure 2).

**Figure 8:**
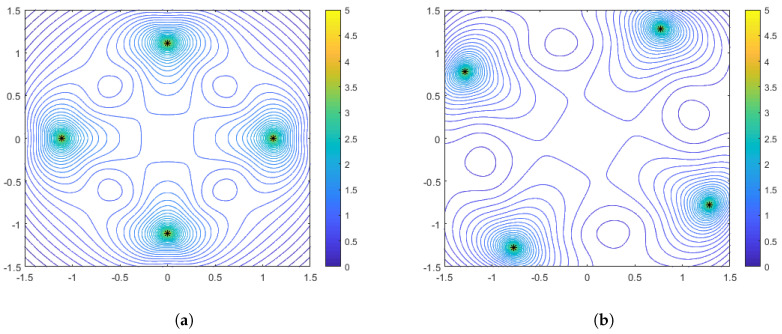
Optimal configurations for N=4 bearing-only sensors with no distance-dependent noise (γ=0). Color contours represent the DFIM values from Equation (20) plotted over various values of source location θ. The left plot (**a**) is for an aggregation parameter of α=−1, and the right plot (**b**) is for an aggregation parameter of α=−10.

**Figure 9:**
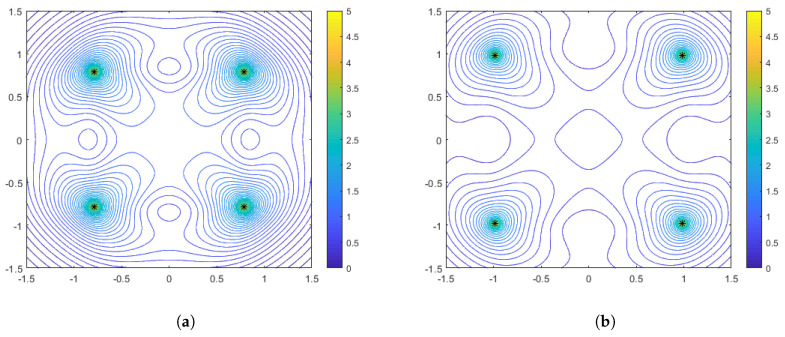
Optimal configurations for N=4 bearing-only sensors with moderate distance-dependent noise (γ=0.1). Color contours represent the DFIM values from Equation (20) plotted over various values of source location θ. The left plot (**a**) is for an aggregation parameter of α=−1, and the right plot (**b**) is for an aggregation parameter of α=−10.

**Figure 10:**
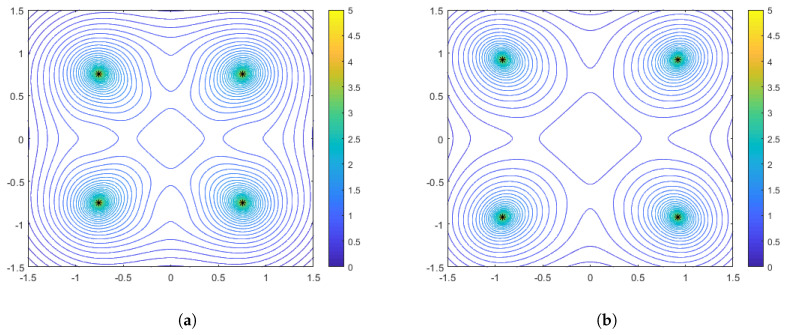
Optimal configurations for N=4 bearing-only sensors with strong distance-dependent noise (γ=0.5). Color contours represent the DFIM values from Equation (20) plotted over various values of source location θ. The left plot (**a**) is for an aggregation parameter of α=−1, and the right plot (**b**) is for an aggregation parameter of α=−10.

As in the range-only sensors case, it is also instructive to plot some particular configurations with accompanying performance surfaces, which correspond to particular points on the spacing measure curves. Figure 8 shows the optimal configurations corresponding to γ=0 (no distance-dependent noise) for aggregation parameter values of α=−1 and α=−10. Interestingly, we see that in this case the optimal configuration for the harmonic mean (α=−1) is a diamond, while for α=−10 the configuration both rotates and spreads out in order to flatten the performance surface. Recall that in the range-only case, the configurations were limited to the largest diamond shape that could meet the constraints. In Figure 9 and Figure 10, we show the optimal sensor configurations for the same aggregation parameter choices, but with γ=0.1 (moderately strong) and γ=0.5 (strong) distance-dependent noise, respectively. As mentioned earlier, all configurations are box-shaped and, as the distance dependence gets stronger, the spacing of the configurations decrease, with the phenomenon being more prevalent in the α=−10 case. Generally, the harmonic mean (α=−1) appears to be a better choice if the practitioner wants to be robust to uncertainty with respect to a priori knowledge of the distance-dependent noise.

### 3.3. Summary of Numerical Results

In the previous sections, we developed two examples, a range-only sensor placement problem and a bearings-only sensor placement problem. These examples were constructed to facilitate an investigation of the effects on optimal sensor configurations when the numerical objectives consisted of different aggregation functions that aggregate the D-optimality over a range of plausible source locations. We calculated the optimal sensor configurations in these examples for varying noise conditions, with the results illustrating both the pattern/shape of the optimal sensor configurations as well as the size of the configuration. Features of the performance were shown for select optimal configurations (for specific α) by plotting contours of the D-optimality measure evaluated at all the possible source points relative to a given optimal configuration, what we refer to as a “performance surface.” While it is well established that larger values of D-optimality correspond to a smaller lower bound on the localization uncertainty, the D-optimality values alone are not easily interpreted. That said, since the D-optimality is the determinant of Fisher information and the associated covariance matrix is just the inverse of the FIM, then a simple approximation of the lower bound of error (in *x* and *y*) can be found by taking $1/\sqrt{DFIM}$ over the span of source points. Hence, the optimization measures presented can directly quantify the source localization uncertainty within the region of interest and gain further insight on what it means to optimize a sensor configuration for select choices of α. Ultimately, the novelty of this work is in considering various choices of aggregation to utilize as an objective for optimization and what the implications are to the resulting optimal sensor placements.

Consider the results from Section 3.1 for the case with range-only sensors. The results associated with Figure 3b, Figure 4b, and Figure 5b plot the D-optimality over the range of plausible source locations under strong distance-dependent noise at α=1, α=−1, and α=−10, respectively. Converting the D-optimality numbers to an average uncertainty gives further perspective on what it means to optimize for the arithmetic mean versus geometric mean versus an even smaller value (more negative) of α. The average variance spans values from 0.3 to 1.2 for α=1, from 0.36 to 0.9 for α=−1, and from 0.5 to 0.76 for α=−10. Choosing the arithmetic mean (α=1) as the aggregation objective in such a strong distance-dependent environment can be a good choice if the variances across the board are viewed as large and we just want to obtain good coverage where we can, i.e., salvage what we can when in a difficult environment. Conversely, the other extreme of α=−10 can be a good choice if the maximal variance of 0.76 is viewed as acceptable; thus, we can cover the entire area with reasonable accuracy and avoid having a portion of the region without adequate coverage. Finally, the harmonic mean (α=−1) could be a good choice as a compromise between these two extremes, where it has the best median accuracy while avoiding the extremes, for good and for bad. The results of these examples demonstrate to practitioners how the planning of optimized sensor configurations can consider these tradeoffs.

## 4. Conclusions

This paper illustrates the impact of aggregation function choice on optimal sensor network configurations for source localization across two sensor schemes, each exhibiting varying degrees of influence from distance-dependent noise on the optimal configuration. We demonstrate a connection between the aggregation function (used to combine information from multiple source hypotheses) and the optimal sensor configuration in all cases. The importance of this connection is that the amount of distance-dependent noise in a particular application will have an impact on how the aggregation selection affects performance. The results of this paper show the strengths of these different effects, so developers can decide where to focus their efforts in choosing what to include in optimal configuration decisions. Future work will include extensions into tracking applications and sparse sensor fields.

## Data Availability

No new data were created or analyzed in this study. Data sharing is not applicable to this article.
